# Demographics, comorbidities, and laboratory parameters in hospitalized patients with SARS-CoV2 infection at a community hospital in rural Pennsylvania

**DOI:** 10.1371/journal.pone.0267468

**Published:** 2022-04-27

**Authors:** Vrushali Pachpande, Sri Harsha Vardhan Senapathi, Karen Williams, Seungwoo Chai, Shobha Mandal, Sheela Prabhu

**Affiliations:** 1 Department of Internal Medicine, Guthrie Medical Group, Sayre, PA, United States of America; 2 Donald Guthrie Foundation, Sayre, PA, United States of America; 3 Department of Surgery, Guthrie Medical Group, Sayre, PA, United States of America; 4 Department of Pharmacy, Guthrie Robert Packer Hospital, Sayre, PA, United States of America; Carol Davila University of Medicine and Pharmacy: Universitatea de Medicina si Farmacie Carol Davila, ROMANIA

## Abstract

**Introduction:**

Inherent differences as well as health disparities among rural and urban populations warrant further studies focused on the characteristics and outcomes in COVID-19 patients in a rural setting. The aim of this study was to describe these elements in patients infected with SARS-CoV2, hospitalized at a single center in rural Pennsylvania.

**Methods:**

Patients with SARS-CoV2 infections hospitalized between March-December 2020 were studied. Data were obtained from electronic health records generated reports and was retrospectively analyzed. Patients were classified into three groups according to severity. Distribution of variables was studied among these three groups. Using certain variables, we ran logistic regression analysis to study the odds of death and requirement of mechanical ventilation (MV).

**Results:**

Among 335 hospitalized patients infected with SARS-CoV2, age more than 65 years increased the severity of clinical status and in-hospital mortality. Gender did not affect odds of death nor need for MV. Hypertension was the most common comorbidity, but diabetes mellitus and chronic obstructive pulmonary disease (COPD) increased the risk of death. In terms of laboratory parameters, our data suggests that maximum LDH marginally increased the risk of death and maximum WBC marginally increased the risk of need for MV and death.

**Conclusion:**

Through our basic analysis of various characteristics of SARS-CoV2 positive patients admitted in a rural hospital, we have identified certain risk factors associated with severe disease and increased in-hospital mortality. These were found to be largely similar to current literature from studies in urban populations, bolstering the reproducibility and generalizability of existing knowledge. This information lays the foundation for future studies to investigate the role of these factors in morbidity and mortality associated with COVID-19 in depth.

## Introduction

COVID-19, disease caused by a novel coronavirus has rapidly evolved into a pandemic since a cluster of cases were identified in Wuhan, China in December 2019 [1]. United States (US) reported the first case in the country in January 2020 at Snohomish County in Washington [2]. As of March 1, 2022, the total number of cases in the US alone has reached 78,900,375 or 24,071.5 cases per 100,000 people [3].

Identifying the characteristics of population at risk of severe outcomes and mortality has public health implications and can inform interventions for disease control, risk stratification and resource allocation. Several studies have evaluated the impact of demographics on COVID-19 related mortality. Most of these are focused on large geographic distributions and urban populations. Health disparities exist between urban and rural populations. As the average age of adults residing in rural areas in the US is higher [4] with significantly greater number of comorbidities [5], and with barriers to care access [6], impact of COVID-19 on rural population may be different than urban population.

Guthrie Robert Packer Hospital (RPH) is a not-for-profit community teaching hospital and an entity under The Guthrie Clinic (TGC). RPH is located in Sayre, Pennsylvania (PA) and is a 254-bed tertiary care hospital that serves the Southern Tier region of New York (NY) and the Northern Tier region of PA. The primary service area for RPH includes Bradford County, Tioga County of PA and Chemung County, Tioga County of NY. According to the Community Health Needs Assessment report in February 2019, among the population served by RPH, 92% is White, non-Hispanic and 20% more than 65 years of age. All four counties have higher rates of obese and overweight individuals compared to national average [7]. These demographic and health related differences contribute to the uniqueness of our patient population.

In this study based in the rural northeast PA region of the US, we have characterized the demographics, comorbidities burden and laboratory abnormalities of hospitalized patients infected with severe acute respiratory syndrome coronavirus 2 (SARS-CoV2) with an aim to identify the independent risk factors associated with severity and mortality.

## Methods

This was a retrospective observational cohort study conducted at RPH. Study period was from March 2020 (when the first inpatient admission related to COVID-19 was noted in our hospital) till December 31, 2020. Hospitalized patients with SARS-CoV2 infection confirmed with a positive reverse transcription polymerase chain reaction (RT-PCR) before or after 14 days of admission date were our study subjects. A cut-off of 14 days was chosen to grossly account for the duration of symptomatic illness and incubation period of COVID-19. As the PCR may be negative in early incubation period, 14 days after the admission was included [8]. Only those who had an outcome by December 31st, 2020, were included in the study. Outcome was defined as a record of discharge from the hospital or in-hospital death. Patients with an incidental finding of SARS-CoV2 infection were included. Patients less than 18 years of age were excluded. Patients transferred to sister hospitals within 24 hours of presentation to the emergency department due to lack of bed availability in our hospital or patients transferred to quaternary care hospitals for specialized services that were unavailable in our hospital were also excluded. In case of patients who had readmissions during the study period, only index hospitalization was considered.

The Institutional Review Board (IRB) of the Guthrie Clinic (IRB00000918) approved the study. As this was a retrospective study with no more than minimal risk to the subjects, a request to waive the informed consent was approved by IRB. Waiving the informed consent is not expected to adversely affect the rights and welfare of the subjects.

Data were collected in the form of an electronic health record (EHR) generated (Epic) reports. Data points included demographic information, pregnancy status, comorbidities, smoking history, laboratory parameters, and certain treatment modalities. Demographic information included age, gender, and race. Age was categorized as ‘less than 65’ for patients less than 65 years and ‘65 and above’ for patients 65 years and above. Comorbidities included were diabetes mellitus (DM), hypertension, chronic obstructive pulmonary disease (COPD), congestive heart failure (CHF), chronic kidney disease (CKD). Laboratory parameters included: hemoglobin, white blood cell count (WBC), lymphocyte count, platelet count, LDH, ferritin, CRP, D-dimer, on admission and during the hospitalization. Treatment characteristics included baseline supplemental oxygen use, transfer to intensive care unit (ICU), need for mechanical ventilation (MV), length of stay and disposition from index admission.

Patients were categorized into non-severe, severe, and critical groups based on their worst clinical status during the hospitalization. We defined non-severe as patients who did not need more oxygen than at their baseline, severe as those who needed any kind of supplemental oxygen exceeding their baseline need, and critical as those who were admitted to the ICU or required MV. These groups were based on the National Institute of Health (NIH) guidelines with an assumption that presence of pneumonia will cause hypoxia and need for supplemental oxygen exceeding baseline oxygen need [9].

Admitted patients were managed according to local guidelines that advised use of remdesivir and dexamethasone in patients with hypoxia (SpO2 less than 94%) or requiring more than baseline oxygen supplementation.

We measured the differences among our three pre-defined severity groups in demographics, hospital length of stay, comorbidities, and laboratory parameters. All continuous variables were compared using Kruskal Wallis H-test/Analysis of Variance and categorical variables using chi-square test statistic.

We ran univariate and multivariate logistic regression to calculate the odds the death and odds of patient needing MV using variables that are shown to have an association with mortality, need for MV and/or severity in current COVID-19 literature. Variables with non-significant difference among the three groups in univariate analysis were still included in multivariate analysis due to our small sample size. Variables chosen were: age category, gender, BMI category, comorbidities, smoking status, laboratory parameters during the course of hospitalization (minimum hemoglobin concentration, maximum WBC count, minimum lymphocyte count, minimum platelet count, maximum ferritin, maximum LDH count, maximum D-Dimer, and maximum CRP) and patient’s treatment status with remdesivir and corticosteroids (CS). For the regression analysis we used LDH values divided by 10 to interpret the change in odds for every 10-unit change in LDH value. Some variables had missing data; this is reported in S1 Table. Observations with missing data were excluded from the regression models. All statistical analysis was done on RStudio v4.0.2 [10].

The Strengthening the Reporting of Observational Studies in Epidemiology (STROBE) guidelines for reporting observational studies were followed [11].

## Results

**Fig 1:**
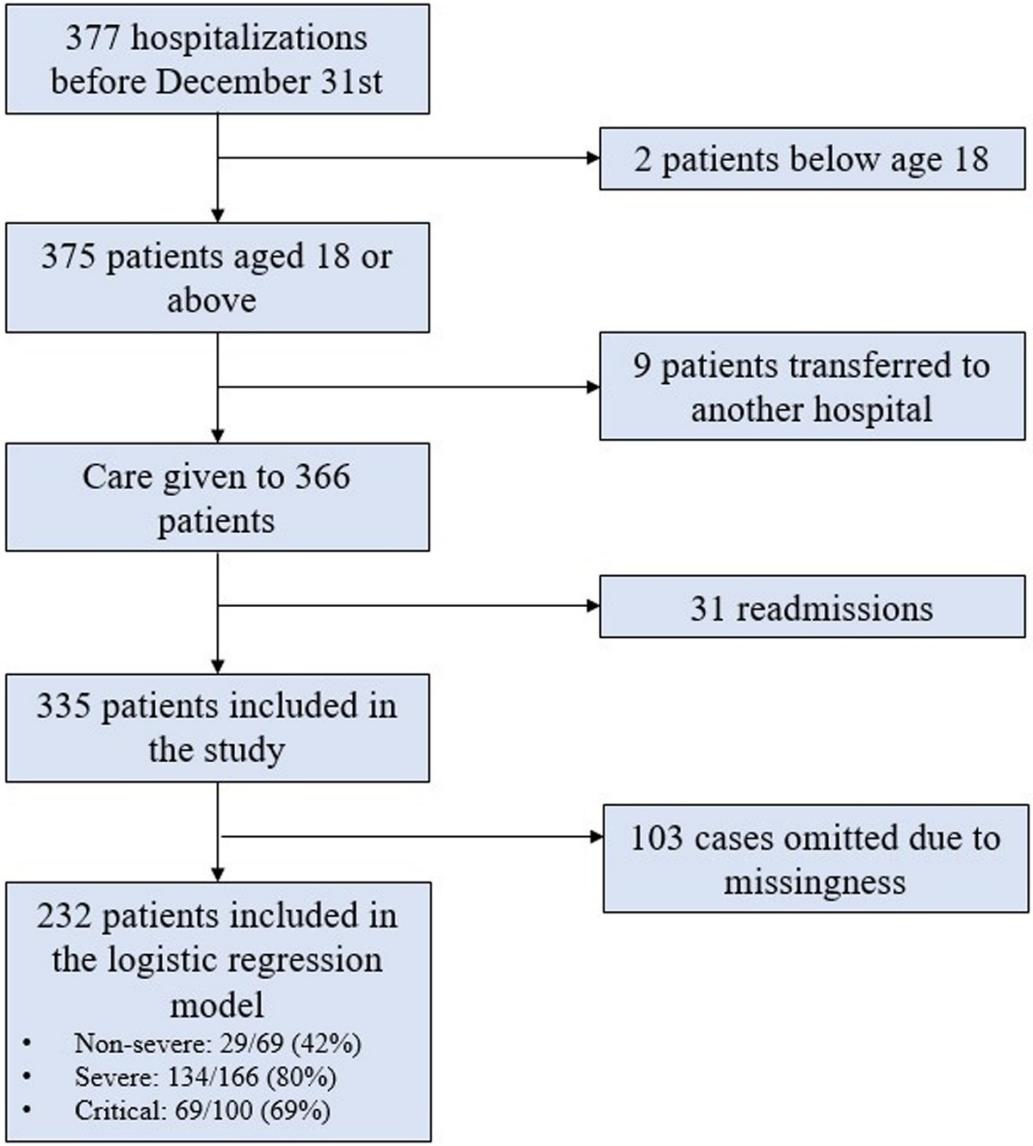
Consort diagram.

There was a total of 335 patients included in this study after the application of inclusion and exclusion criteria as stated above. Out of these, 69 patients were in non-severe group, 166 patients in severe group and 100 patients in critical group. The logistic regression analysis was conducted on 232 patients after removing observations with missing values. Of the 232 patients included in the logistic regression, 29 were non-severe cases, 134 were severe and 69 were in critical group (Fig 1).

Table 1 shows demographics and comorbidities between these groups. The median age of patients being admitted was 72 years with significant differences between the three groups. The median length of hospital stay was 6 days. Non-severe patients tended to have shorter stays and critical patients had longer stays with median 7.5 days and IQR 10.2 days. In non-severe and severe groups, 46.4% and 48.2% patients were male respectively. However, in critical group, 64% of patients were male and this proportion was significantly different compared to the non-severe and severe groups. The median BMI of the study population was 31.2 kg/m2 and 95.8% of all patients were Caucasians. Median BMI tended to increase according to the severity, this difference did not reach statistical significance. History of smoking (either current or former) was noted in 51.9% of all patients. Although percentages of patients with smoking history were noted to increase according to severity, this difference was not statistically significant. Most common comorbidity was hypertension which was present in 69% of the patients followed by DM, noted in 43% of the patients. Higher prevalence of DM was noted in critical patients (54%) compared to non-severe patients (29%). Overall in-hospital mortality was 19.4%, and it was highest in critical patients at 51%. Total in-hospital mortality was 6/29 (20%) before and 59/306 (19%) after RECOVERY trial results were released on June 16, 2020.

**Table 1:** Demographics, comorbidities, vital status, and treatment modalities between the three severity groups.

	Non-severe (n (%))	Severe (n (%))	Critical (n (%))	Total	p
Sample size (n)	69	166	100	335	
Age ^a^ *	68.0 (56–79)	73.0 (65–81)	72.5 (64–79)	72.0 (63–80)	0.037
Age Category *					
• 65 and above	40 (58.0)	125 (75.3)	74 (74.0)	239 (71.3)	0.022
• Less than 65	29 (42.0)	41 (24.7)	26 (26.0)	96 (28.7)	
BMI^a^	29.2 (24.5–39.8)	31.4 (27.69–38.05)	32.0 (26.98–37.75)	31.2 (26.96–37.73)	0.183
BMI category					
• Normal	15 (21.7)	28 (16.9)	11 (11.0)	54 (16.1)	0.251
• Overweight	15 (21.7)	36 (21.7)	27 (27.0)	78 (23.3)	
• Obese	17 (24.6)	63 (38.0)	43 (43.0)	123 (36.7)	
• Severe Obesity	9 (13.0)	30 (18.1)	15 (15.0)	54 (16.1)	
• Missing	13(18.8)	9(5.4)	4(4.0)	26(7.8)	
Hospital LOS ^a^*	4.0 (2–7)	7.0 (5–10)	7.5 (4–14.25)	6.0 (4–10)	<0.001
Gender*					
• Female	37 (53.6)	86 (51.8)	36 (36.0)	159 (47.5)	0.023
• Male	32 (46.4)	80 (48.2)	64 (64.0)	176 (52.5)	
Pregnancy	5 (7.2)	0 (0)	0 (0)	5 (1.5)	<0.001
Race					
• Black or African American	2 (2.9)	4 (2.4)		6 (1.8)	0.447
• Patient Refused	1 (1.4)		1 (1.0)	2 (0.6)	
• Unknown	1 (1.4)	1 (0.6)	2 (2.0)	4 (1.2)	
• White	65 (94.2)	159 (95.8)	97 (97.0)	321 (95.8)	
• Asian		2 (1.2)		2 (0.6)	
Smoking status ^c^	30 (43.5)	88 (53.0)	56 (56.0)	174 (51.9)	0.257
Diabetes*^c^	20 (29.0)	70 (42.2)	54 (54.0)	144 (43.0)	0.005
HTN ^c^	44 (63.8)	122 (73.5)	65 (65.0)	231 (69.0)	0.202
COPD ^c^	10 (14.5)	32 (19.3)	14 (14.0)	56 (16.7)	0.459
CHF ^c^	17 (24.6)	49 (29.5)	22 (22.0)	88 (26.3)	0.379
CKD ^c^	14 (20.3)	40 (24.1)	23 (23.0)	77 (23.0)	0.819
Vital status* (Expired)	1 (1.4)	13 (7.8)	51 (51.0)	65 (19.4)	<0.001
Remdesivir* ^t^	11 (15.9)	129 (77.7)	65 (65.0)	205 (61.2)	<0.001
Steroid Use* ^t^	20 (29.0)	139 (83.7)	84 (84.0)	243 (72.5)	<0.001

Data is presented as number of patients (%).

^a^: Non-normal distributions presented as median [IQR].

* P <0.05, Statistically significant. Abbreviations: BMI: Body Mass Index; HTN: Hypertension; COPD: Chronic Obstructive Pulmonary Disease; CHF: Congestive Heart Failure; CKD: Chronic Kidney Disease.

^c^: Prevalence of comorbidity in each severity group.

^t^: Percentage of patients treated with drug. Vital status (Expired) stands for death.

Comparison of laboratory factors among the groups is shown in Table 2. Inflammatory markers like LDH, ferritin, CRP and D-dimer tended to be significantly different according to the severity of the disease. The difference was noted as early as day 1 of admission. Patients in critical group had significantly higher WBC and lower lymphocyte counts compared to other groups, this was noted on day 1 of presentation as well as during the hospital stay.

**Table 2:** Laboratory parameters between the three severity groups.

Variable (units) [normal range]	Non-severe	Severe	Critical	p
	Median (IQR)	Median (IQR)	Median (IQR)	
LDH day 1* (U/L) [313–618 U/L]	286.0 (213–352)	319.0 (245–432)	417.0 (298.25–595.5)	<0.001
Max LDH* (U/L) [313–618 U/L]	298.5 (232–339.25)	360.0 (272.25–478)	517.0 (351–737.5)	<0.001
Ferritin day 1* (NG/ML) [18.0–464.0]	364.0 (144.5–695.25)	411.0 (204.75–780.25)	688.5 (368.75–1606.75)	0.002
Max Ferritin* (NG/ML) [18.0–464.0]	389.0 (171–585.5)	514.5 (279.25–1013.75)	855.0 (441–1635)	<0.001
CRP day 1* (mg/dl) [<0.5]	2.9 (1.65–4.57)	4.3 (2.28–9.17)	9.7 (3.59–17.66)	<0.001
Max CRP* (mg/dl) [<0.5]	3.1 (1.87–5.49)	5.6 (2.31–9.70)	14.6 (7.25–21.91)	<0.001
D-Dimer day 1* (UG/ML) [0–6.5]	1.0 (0.54–1.81)	1.1 (0.70–2.01)	2.0 (0.90–3.96)	
Max D-Dimer* (UG/ML) [0–6.5]	1.0 (0.54–1.79)	1.2 (0.66–2.24)	3.0 (1.53–5.75)	<0.001
Hemoglobin day 1 (g/dL) [13.7–17.5]	13.1 (11.35–14.40)	13.0 (11.4–14.4)	12.7 (10.58–14.0)	0.275
Min Hemoglobin* (g/dL) [13.7–17.5]	12.0 (10.2–12.9)	11.6 (10.3–13.2)	10.2 (8.08–12)	<0.001
WBC day 1* (K/uL) [4.23–9.07]	6.7 (5.06–9.78)	6.9 (5.20–9.63)	10.7 (7.2–16.34)	<0.001
Max WBC* (K/uL) [4.23–9.07]	7.4 (5.60–9.88)	9.8 (7.59–13.51)	15.9 (10.94–21.7)	<0.001
Min WBC* (K/uL) [4.23–9.07]	4.5 (3.73–6.12)	5.1 (3.65–6.69)	6.7 (4.67–10.21)	<0.001
Platelets day 1 (K/uL) [163–337]	203.0 (169.5–248.5)	194.0 (158–243)	190.5 (157–253.3)	0.84
Min platelet* (K/uL) [163–337]	180.0 (138.5–272.5)	165.0 (133–207)	153.5 (106.5–193.5)	0.005
Lymphocytes day* 1 (K/uL) [1.32–3.57]	1.3 (0.89–1.82)	1.0 (0.72–1.48)	1.0 (0.62–1.55)	0.02
Min lymphocytes* (K/uL) [1.32–3.57]	0.9 (0.63–1.22)	0.6 (0.42–0.91)	0.5 (0.29–0.68)	0.001

Abbreviations: min: Minimum; max: Maximum; LDH: Lactate dehydrogenase; CRP: C-reactive protein; WBC: white cell count

*: Laboratory parameters with significant difference between any two severity groups at 95% level of significance.

Tables 3 and 4 show the effect of comorbidities and laboratory parameters on the odds of death and MV, respectively. In the univariate and multivariate logistic regression for odds of death, age category, COPD, DM, maximum LDH and maximum WBC significantly affected mortality. Patients with DM were 9.48 times more likely to die compared to patients who were not diabetic with a 95% confidence interval (CI) of (2.85–37.48) when adjusted for other factors. Having COPD increased the odds of death by 5.24 times with a CI of 1.26–22.91 when adjusted for other factors. CS use was associated with higher odds of death. Maximum WBC marginally increased the odds of requiring MV. Hypertension and low hemoglobin appeared to be protective of need for MV.

**Table 3:** Multivariate logistic regression calculating odds of death for each variable.

Variable	Multivariate Odds Ratios (95% CI, p-value)
***Age category (Less than 65)*** *	0.04 (0.00–0.29)
***Gender (Male)***	
***BMI Category***	
Normal	-
Overweight	-
Obese	-
Severe Obesity	-
***Smoking status (Yes)*** ^***c***^** **	-
***COPD (Present)*** ^***c*,**^ *** **	5.24 (1.26–22.91)
***Diabetes (Present)*** ^***c*,**^ *** **	9.48 (2.85–37.48)
***HTN (Present)*** ^***c***^** **	-
***CHF (CHF)*** ^***c***^** **	-
***CKD (CKD)*** ^***c***^** **	-
***Max Ferritin***	-
***Min Hemoglobin***	-
***Min Platelets***	-
Max LDH*, **	1.01 (1.00–1.01)
***Max D-Dimer***	-
***Minimum lymphocyte count***	-
***Max WBC*** *	1.14 (1.06–1.23)
***Max CRP***	-
Steroid Use* ***(Yes)*** ^***t***^** **	24.35 (2.67–318.19)
R***emdesivir (Yes)*** ^***t***^** **	-

Abbreviations: min: Minimum; max: Maximum; BMI: Body Mass Index; HTN: Hypertension; COPD: Chronic Obstructive Pulmonary Disease; CHF: Congestive Heart Failure; CKD: Chronic Kidney Disease; LDH: Lactate dehydrogenase; CRP: C-reactive protein; WBC: white cell count; c: Odds of dying among patients with comorbidities compared with no comorbidity. t: Odds of dying among patients with treatment compared with no treatment.

*: Variables significantly affecting the odds of death

**: LDH values were divided by 10 and the odds of death should be interpreted for every 10 unit increase in LDH values.

**Table 4:** Multivariate logistic regression calculating odds of MV for each variable.

	Multivariate Odds Ratios (95% CI, p-value)
***Age category (Less than 65)***	-
***Gender (Male)***	-
***BMI Category***	
Normal	-
Overweight	-
Obese	-
Severe Obesity	-
***Smoking status (Yes)*** ^***c***^** **	-
***COPD (Present)*** ^***c***^** **	-
***Diabetes (Present)*** ^***c***^** **	-
HTN* ***(Present)*** ^***c***^** **	0.09 (0.01–0.67)
***CHF (CHF)*** ^***c***^** **	-
***CKD (CKD)*** ^***c***^** **	-
***Max Ferritin***	-
***Min Hemoglobin*** *	0.71 (0.49–0.97)
***Min Platelets***	-
Max LDH*^,^ **** **	-
***Max D-Dimer***	-
***Minimum lymphocyte count***	-
***Max WBC*** *	1.31 (1.17–1.53)
***Max CRP***	-
***Steroid Use (Yes)*** ^***t***^** **	-
R***emdesivir (Yes)*** ^***t***^** **	-

Abbreviations: min: Minimum; max: Maximum; BMI: Body Mass Index; HTN: Hypertension; COPD: Chronic Obstructive Pulmonary Disease; CHF: Congestive Heart Failure; CKD: Chronic Kidney Disease. c: Odds of MV among patients with the comorbidity compared with no comorbidity. t: Odds of MV among patients with treatment compared with no treatment.

*: Variables significantly affecting the odds of MV

**: LDH values were divided by 10 and the odds of MV should be interpreted for every 10 unit increase in LDH values.

## Discussion

Our study has corroborated current knowledge that older adults with COVID-19 are at increased risk of unfavorable outcomes. In a report from Chinese CDC, although an overall case fatality rate (CFR) was 2.3%, age specific CFR was much higher in older patients; 8% in those aged 70–79 years and 14.8% in those aged 80 and older [12]. In a study from Italy, authors observed increasing CFR in older patients, with 1% in patients between 50–59 years and 20.2% in those aged 80 years or older [13]. A large cohort study from UK showed increasing age to be strongly associated with risk of COVID-19 related death, with people aged 80 or over having a more than 20-fold-increased risk compared to 50–59-year-olds [14]. Similar trends of increased mortality among older patients have been shown among hospitalized patients. In a study of 5700 patients hospitalized in NY area, higher mortality was seen among patients more than 65 years of age compared to those between 18–65 years irrespective of need for MV [15]. More recently, a large cohort study by Nguyen et al. showed that hospital mortality increased in association with increasing age [16].

In our study, although more males tended to be in critical group, gender did not affect the odds of death nor MV in the regression analysis. Shah et al. reported observations from hospitalized COVID-19 patients in rural Southwest Georgia [17]. In their study, female gender was associated with reduction in in-hospital-mortality. A prospective observational study from Italy did not find gender-based difference in in-hospital mortality [18]. A systematic review and meta-analysis Liid et al. found association of male gender with increased severity of COVID-19 [19].

A likely explanation of increasing mortality with age is the higher burden of comorbidities in older patients. Hypertension is the most common comorbidity among our studied patients. We noted DM and COPD to have independently increased the odds of death. Systematic review and meta-analysis by Mudatsir et al. identified comorbidities associated with a higher risk of severe disease, these included chronic respiratory disease, hypertension, diabetes mellitus and cardiovascular disease [20]. In a metanalysis by Kumar et al., diabetics were found to have a two-fold increase in mortality as well as severity of COVID-19, as compared to non-diabetics [21]. Meta-analysis by Huang et al. showed that DM was associated with mortality, severe COVID-19, acute respiratory distress syndrome (ARDS), and disease progression in patients with COVID-19 [22]. The impact of patterns of glycemic control in diabetics on this effect on mortality and severity remains to be explored. In a recent nationwide study from Korea, COPD independently increased mortality among COVID-19 patients [23].

We studied the associations of the hematological parameters on admission and through the hospital stay with severity, and noted that minimum hemoglobin, maximum WBC, minimum lymphocyte counts, and minimum platelet counts were associated with higher severity. These findings are in alignment with the report by Rahman et al. [24]. Nadir platelet counts did not affect the odds of death significantly, as shown in Table 3. Hypertension and minimum hemoglobin reduced the odds of requiring MV (Table 4), however, this should be cautiously interpreted as other factors affecting MV like do-not-resuscitate/do-not-intubate status, goals of care discussions and forgoing MV, were not considered in our study. Factors like ethnicity, stage of hypertension, degree of control of blood pressure, impact of specific classes of antihypertensive drugs and degree of anemia may affect these results [25, 26].

The inflammatory markers were noted to be higher in more severe groups and this difference was apparent on day 1 of presentation. Maximum LDH level was shown to be an independent risk factor, marginally increasing the odds of death. In a study done in the emergency department of Italy, elevated LDH was found to have an inverse relation with the respiratory performance and was an independent risk factor for the severity and mortality of COVID-19 [27].

Dexamethasone was inconsistently used in our patients with COVID-19 prior to the news release of RECOVERY on June 16, 2020. After these results were available, local guidelines advised use of remdesivir and dexamethasone if patients had hypoxia with SpO2 less than 94% or requiring more than baseline oxygen supplementation. Thus, adherence to the guidelines may explain correlation, not causation, of CS use with severe disease and death raising the possibility of spurious association. CS use may be a proxy for a more severe patient status instead of being an actual risk factor for death. However, there are several limitations in interpreting this finding. Post-hoc data from RECOVERY showed that the timing of CS therapy in treatment of COVID-19 may affect outcomes. Dexamethasone treatment was associated with a reduction in 28-day mortality among those with symptoms for more than 7 days but not among those with a symptom onset less than 7 days [28]. Data by Keller et al. showed that early CS in patients with low CRP (<10 mg/dl) was associated with significantly increased risk of mortality or MV [29]. A recently published prospective multicentric study showed an independent association of CS use in critically ill elderly (70 years and older) COVID-19 patients with increased mortality [30]. Our data included older patient population with median age of 72 years and 71.3% patients above the age of 65 years. As we did not include factors like pre-hospitalization use of CS, specifics of CS use (timing, type, dose, or duration) during hospitalization, degree of inflammatory response and days since onset of symptoms, no definite conclusions can be drawn related to the effects of CS use on severity or mortality. The population served by RPH includes older patients with higher number of comorbidities and more patients in overweight and obese categories. Increasing age, obesity and burden of comorbidities are known risk factors for severe COVID-19 disease and this potentially explains the higher in-hospital mortality rate. Factors such as access to care, attitudes of population in rural settings are expected to impact thresholds of hospitalizations, in turn affecting severity at the time of admission and in-hospital mortality.

### Limitations

We acknowledge the limitations of our study. The data extraction was done via discrete data fields from the EHR. This precludes the precision obtained through a manual review. Situations of temporary oxygen need not discretely recorded may have been missed by the analysis therefore would result in incorrect classification of patients as non-severe. Unequal distribution of the missing data among the severity groups as reported in S1 Table has a potential to affect the results. We have included all patients with a positive RT-PCR for SARS-CoV2. Thus, patients admitted for reasons other than COVID-19 and incidentally found SARS-CoV2 positive patients were included. The role of socioeconomic status in disease prevalence and progression was beyond the scope of this study. The specifics of treatment regimen of remdesivir (duration) and CS (timing, type, dose, and duration) were not studied. Impact of new variants of concern and vaccination against SARS-CoV-2 limit the generalizability of the data. Discharge disposition may impact length of stay, this was not studied. The clinical capacity of our center, criteria for upgrading from one setting to the other and other health disparities specific to our population may affect the external validity of the data and are beyond the scope of this study.

## Conclusion

We have summarized our findings of a preliminary study broadly focused on various aspects of the disease in our patient population. Our study serves to validate current knowledge regarding severity and mortality related to COVID-19 in a rural US population. This is aimed to lay the groundwork for an in-depth scrutiny of identified risk factors as our experience and knowledge of this novel disease deepens.

## Supporting information

S1 Table Missing data from the variables in the laboratory parameters in Table 2 in each severity groups. (DOCX)

S2 Table Univariate and Multivariate Logistic regression calculating odds of death for each variable. (DOCX)

S3 Table Univariate and Multivariate Logistic regression calculating odds of ventilation for each variable. (DOCX)

## References

[1] WHO Director-General’s remarks at the media briefing on 2019-nCoV on 11 February 2020 [Internet]. [cited 2021 Jun 9]. Available from: https://www.who.int/director-general/speeches/detail/who-director-general-s-remarks-at-the-media-briefing-on-2019-ncov-on-11-february-2020

[2] Holshue ML, DeBolt C, Lindquist S, Lofy KH, Wiesman J, Bruce H, et al. First Case of 2019 Novel Coronavirus in the United States. N Engl J Med. 2020 Mar 5;382(10):929–36. doi: 10.1056/NEJMoa2001191 32004427 PMC7092802

[3] COVID Data Tracker Weekly Review | CDC [Internet]. [cited 2021 May 18]. Available from: https://www.cdc.gov/coronavirus/2019-ncov/covid-data/covidview/index.html

[4] USDA ERS—Rural America at a Glance, 2019 Edition [Internet]. [cited 2021 Jun 9]. Available from: https://www.ers.usda.gov/publications/pub-details/?pubid=95340

[5] National Center for Health Statistics. Health, United States, 2017: With Special Feature on Mortality. U.S. Department of Health & Human Services. 2017. 30702833

[6] Healthcare Access in Rural Communities Introduction—Rural Health Information Hub [Internet]. [cited 2021 Jun 9]. Available from: https://www.ruralhealthinfo.org/topics/healthcare-access

[7] CHNA RPH [Internet]. [cited 2021 Jun 9]. Available from: https://www.guthrie.org/sites/default/files/PDFs/about-us/Robert-Packer-Hospital-CHNA-Final.pdf

[8] Zaki N, Mohamed EA. The estimations of the COVID-19 incubation period: A scoping reviews of the literature. J Infect Public Health. 2021 May 1;14(5):638–46. doi: 10.1016/j.jiph.2021.01.019 33848893 PMC7869687

[9] Clinical Spectrum | COVID-19 Treatment Guidelines [Internet]. [cited 2021 Dec 21]. Available from: https://www.covid19treatmentguidelines.nih.gov/overview/clinical-spectrum/

[10] Bunn A, Korpela M. A language and environment for statistical computing. Found Stat Comput. 2013;2:1–12.

[11] Vandenbroucke JP, Von Elm E, Altman DG, Gøtzsche PC, Mulrow CD, Pocock SJ, et al. Strengthening the Reporting of Observational Studies in Epidemiology (STROBE): Explanation and elaboration. PLoS Med. 2007 Oct;4(10):1628–54. 10.1371/journal.pmed.0040297 PMC2020496 17941715

[12] Wu Z, McGoogan JM. Characteristics of and Important Lessons from the Coronavirus Disease 2019 (COVID-19) Outbreak in China: Summary of a Report of 72314 Cases from the Chinese Center for Disease Control and Prevention. JAMA—J Am Med Assoc. 2020;323(13):1239–42. doi: 10.1001/jama.2020.2648 32091533

[13] Medical Association A. Case-Fatality Rate and Characteristics of Patients Dying in Relation to COVID-19 in Italy. 2020. 10.1001/jama.2020.4683 32203977

[14] Williamson EJ, Walker AJ, Bhaskaran K, Bacon S, Bates C, Morton CE, et al. 3 TPP, Horsforth, UK. 4 NIHR Health Protection Research Unit in Immunisation, London, UK. 5 Intensive Care National Audit and Research Centre (ICNARC). Nature. 2020;584:430. doi: 10.1038/s41586-020-2521-4 32640463 PMC7611074

[15] Richardson S, Hirsch JS, Narasimhan M, Crawford JM, McGinn T, Davidson KW, et al. Presenting Characteristics, Comorbidities, and Outcomes among 5700 Patients Hospitalized with COVID-19 in the New York City Area. JAMA—J Am Med Assoc. 2020;323(20):2052–9. 10.1001/jama.2020.6775 PMC7177629 32320003

[16] Nguyen NT, Chinn J, Nahmias J, Yuen S, Kirby KA, Hohmann S, et al. Outcomes and Mortality among Adults Hospitalized with COVID-19 at US Medical Centers. JAMA Netw Open. 2021;4(3):20–3. doi: 10.1001/jamanetworkopen.2021.0417 33666657 PMC8547263

[17] Shah P, Owens J, Franklin J, Mehta A, Heymann W, Sewell W, et al. Demographics, comorbidities and outcomes in hospitalized Covid-19 patients in rural southwest Georgia. Ann Med. 2020;52(7):354–60. doi: 10.1080/07853890.2020.1791356 32620056 PMC7877969

[18] Redaelli M, Landoni G, Di Napoli D, Morselli F, Sartorelli M, Sartini C, et al. Novel Coronavirus Disease (COVID-19) in Italian Patients: Gender Differences in Presentation and Severity. Saudi J Med Med Sci. 2021;9(1):59. doi: 10.4103/sjmms.sjmms_542_20 33519345 PMC7839571

[19] Liid X, Zhong X, Wang Y, Zeng X, Luo T, Liuid Q. Clinical determinants of the severity of COVID-19: A systematic review and meta-analysis. 2021. 10.1371/journal.pone.0250602 PMC8092779 33939733

[20] Mudatsir M, Karunia Fajar J, Wulandari L, Soegiarto G, Ilmawan M, Purnamasari Y, et al. Predictors of COVID-19 severity: a systematic review and meta-analysis [version 2; peer review: 2 approved]. 2021. 10.12688/f1000research.26186.1 PMC7607482 33163160

[21] Kumar A, Arora A, Sharma P, Anikhindi SA, Bansal N, Singla V, et al. Is diabetes mellitus associated with mortality and severity of COVID-19? A meta-analysis. Diabetes Metab Syndr Clin Res Rev. 2020 Jul 1;14(4):535–45. 10.1016/j.dsx.2020.04.044 PMC7200339 32408118

[22] Huang I, Lim MA, Pranata R. Diabetes mellitus is associated with increased mortality and severity of disease in COVID-19 pneumonia–A systematic review, meta-analysis, and meta-regression: Diabetes and COVID-19. Diabetes Metab Syndr Clin Res Rev. 2020 Jul 1;14(4):395–403. doi: 10.1016/j.dsx.2020.04.018 32334395 PMC7162793

[23] Chul Lee S, Ju Son K, Hoon Han C, Cheol Park S, Ye Jung J. Impact of COPD on COVID-19 prognosis: A nationwide population-based study in South Korea. Sci Reports |. 123AD;11:3735. 10.1038/s41598-021-83226-9 PMC7880985 33580190

[24] Rahman A, Niloofa R, Jayarajah U, De Mel S, Abeysuriya V, Seneviratne SL. (No Title). Am J Trop Med Hyg. 2021;104(4):1188–201. doi: 10.4269/ajtmh.20-1536 33606667 PMC8045618

[25] Chen R, Yang J, Gao X, Ding X, Yang Y, Shen Y, et al. Influence of blood pressure control and application of renin-angiotensin-aldosterone system inhibitors on the outcomes in COVID-19 patients with hypertension. J Clin Hypertens. 2020 Nov 1;22(11):1974–83. doi: 10.1111/jch.14038 33006442 PMC7537535

[26] Oh SM, Skendelas JP, Macdonald E, Bergamini M, Goel S, Choi J, et al. On-admission anemia predicts mortality in COVID-19 patients: A single center, retrospective cohort study. Am J Emerg Med. 2021 Oct 1;48:140–7. doi: 10.1016/j.ajem.2021.03.083 33895645 PMC8007204

[27] Li C, Ye J, Chen Q, Hu W, Wang L, Fan Y, et al. Elevated Lactate Dehydrogenase (LDH) level as an independent risk factor for the severity and mortality of COVID-19. Aging (Albany NY). 2020 Aug 15;12(15):15670–81. doi: 10.18632/aging.103770 32805722 PMC7467395

[28] Low-cost dexamethasone reduces death by up to one third in hospitalised patients with severe respiratory complications of COVID-19—RECOVERY Trial [Internet]. [cited 2021 Jun 9]. Available from: https://www.recoverytrial.net/news/low-cost-dexamethasone-reduces-death-by-up-to-one-third-in-hospitalised-patients-with-severe-respiratory-complications-of-covid-19

[29] Keller MJ, Kitsis EA, Arora S, Chen JT, Agarwal S, Ross MJ, et al. Effect of Systemic Glucocorticoids on Mortality or Mechanical Ventilation in Patients With COVID-19. J Hosp Med. 2020 Aug 1;15(8):489. doi: 10.12788/jhm.3497 32804611 PMC7518134

[30] Jung C, Wernly B, Fjølner J, Bruno RR, Dudzinski D, Artigas A, et al. Steroid use in elderly critically ill COVID-19 patients. Eur Respir J. 2021 Oct 1;58(4). doi: 10.1183/13993003.00979-2021 34172464 PMC8246007

