# Supplementary material for: Demographics, comorbidities, and laboratory parameters in hospitalized patients with SARS-CoV2 infection at a community hospital in rural Pennsylvania
Source: PLoS One. 2022 Apr 27;17(4):e0267468. doi: 10.1371/journal.pone.0267468 (PMC9045616; doi:10.1371/journal.pone.0267468)
Supplement: S1 Table — (DOCX) [file pone.0267468.s001.docx]

**S1 Table:** Missing data from the variables in the laboratory parameters in Table 2 in each severity groups

| **Variable** | **Non-severe** | **Severe** | **Critical** | **Total** |
| --- | --- | --- | --- | --- |
| LDH day 1 | 36 (52.2%) | 31 (18.7%) | 34 (34.0 %) | 101 (30.1%) |
| Max LDH | 31 (44.9%) | 16 (9.6%) | 18 (18.0%) | 65 (19.4%) |
| Ferritin day 1 | 35 (50.7%) | 22 (13.3%) | 32 (32.0%) | 89 (26.6%) |
| Max Ferritin | 30 (43.5%) | 12 (7.2%) | 19 (19.0 %) | 61 (18.2%) |
| CRP day 1 | 34 (49.3%) | 29 (17.5%) | 29 (29.0%) | 92 (27.5%) |
| Max CRP | 60 (17.9%) | 29 (42.0%) | 15 (9.0%) | 16 (16.0%) |
| D-Dimer day 1 | 115 (34.3%) | 38 (55.1%) | 37 (22.3%) | 40 (40.0%) |
| Max D-Dimer | 75 (22.4%) | 33 (47.8%) | 18 (10.8%) | 24 (24.0%) |
| Hemoglobin day 1 | 3 (0.9%) | 2 (2.9%) | 1 (0.6%) | 0 (0%) |
| Min Hemoglobin | 3 (0.9%) | 2 (2.9%) | 1 (0.6%) | 0 (0%) |
| WBC day 1 | 3 (0.9%) | 2 (2.9%) | 1 (0.6%) | 0 (0%) |
| Max WBC | 0(0%) | 0 (0%) | 0 (0%) | 0 (0%) |
| Min WBC | 0(0%) | 0 (0%) | 0 (0%) | 0 (0%) |
| Platelets day 1 | 3 (0.9%) | 2 (2.9%) | 1 (0.6%) | 0 (0%) |
| Min platelets | 3 (0.9%) | 2 (2.9%) | 1 (0.6%) | 0 (0%) |
| Lymphocyte day 1 | 3 (0.9%) | 2 (2.9%) | 1 (0.6%) | 0 (0%) |
| Min lymphocyte | 3 (0.9%) | 2 (2.9%) | 1 (0.6%) | 0 (0%) |
