# Supplementary material for: Demographics, comorbidities, and laboratory parameters in hospitalized patients with SARS-CoV2 infection at a community hospital in rural Pennsylvania
Source: PLoS One. 2022 Apr 27;17(4):e0267468. doi: 10.1371/journal.pone.0267468 (PMC9045616; doi:10.1371/journal.pone.0267468)
Supplement: S2 Table — (DOCX) [file pone.0267468.s002.docx]

**S2 Table:** Univariate and Multivariate Logistic regression calculating odds of death for each variable

| **Variable** | **Univariate Odds Ratios (95% CI, p-value)** | **Multivariate Odds Ratios (95% CI, p-value)** |
| --- | --- | --- |
| ***Age category (Less than 65)**** | 0.20 (0.08-0.45, p<0.001) | 0.04 (0.00-0.29, p=0.004) |
| ***Gender (Male)*** | 1.25 (0.72-2.16, p=0.431) | 1.26 (0.36-4.60, p=0.724) |
| ***BMI Category*** |  |  |
| Normal | - | - |
| Overweight | 1.37 (0.55-3.65, p=0.512) | 0.35 (0.05-2.12, p=0.264) |
| Obese | 1.77 (0.78-4.44, p=0.191) | 0.25 (0.05-1.22, p=0.093) |
| Severe Obesity | 1.00 (0.34-2.94, p=1.000) | 0.19 (0.02-1.55, p=0.140) |
| ***Smoking status (Yes) ^c^*** | 1.50 (0.87-2.62, p=0.149) | 0.85 (0.23-3.06, p=0.801) |
| ***COPD (Present) ^c,^ **** | 1.49 (0.74-2.88, p=0.248) | 5.24 (1.26-22.91, p=0.023) |
| ***Diabetes (Present) ^c,^ **** | 2.18 (1.26-3.81, p=0.006) | 9.48 (2.85-37.48, p=0.001) |
| ***HTN (Present) ^c^*** | 1.02 (0.57-1.86, p=0.957) | 0.51 (0.13-2.11, p=0.339) |
| ***CHF (CHF) ^c^*** | 1.20 (0.65-2.17, p=0.546) | 1.25 (0.37-4.28, p=0.720) |
| ***CKD (CKD) ^c^*** | 1.12 (0.58-2.07, p=0.728) | 1.09 (0.28-4.17, p=0.898) |
| ***Max Ferritin*** | 1.00 (1.00-1.00, p=0.146) | 1.00 (1.00-1.00, p=0.645) |
| ***Min Hemoglobin*** | 0.77 (0.68-0.87, p<0.001) | 0.79 (0.60-1.01, p=0.067) |
| ***Min Platelets*** | 0.99 (0.98-0.99, p<0.001) | 0.99 (0.98-1.00, p=0.006) |
| ***Max LDH*, ***** | 1.00 (1.00-1.00, p<0.001) | 1.01 (1.00-1.01, p=0.001) |
| ***Max D-Dimer*** | 1.22 (1.13-1.33, p<0.001) | 1.07 (0.94-1.23, p=0.293) |
| ***Minimum lymphocyte count*** | 1.00 (1.00-1.00, p=0.359) | 1.00 (1.00-1.00, p=0.832) |
| ***Max WBC**** | 1.15 (1.10-1.20, p<0.001) | 1.14 (1.06-1.23, p<0.001) |
| ***Max CRP*** | 1.10 (1.06-1.14, p<0.001) | 1.01 (0.94-1.09, p=0.818) |
| ***Steroid Use* (Yes) ^t^*** | 5.70 (2.42-16.77, p<0.001) | 24.35 (2.67-318.19, p=0.007) |
| R***emdesivir (Yes) ^t^*** | 1.42 (0.81-2.56, p=0.232) | 0.58 (0.12-3.07, p=0.507) |

Abbreviations: min: Minimum; max: Maximum; BMI: Body Mass Index; HTN: Hypertension; COPD: Chronic Obstructive Pulmonary Disease; CHF: Congestive Heart Failure; CKD: Chronic Kidney Disease; LDH: Lactate dehydrogenase; CRP: C-reactive protein; WBC: white cell count; c : Odds of dying among patients with comorbidities compared with no comorbidity. t: Odds of dying among patients with treatment compared with no treatment. *: Variables significantly affecting the odds of death, **: LDH values were divided by 10 and the odds of death should be interpreted for every 10 unit increase in LDH values.
