# Supplementary material for: Demographics, comorbidities, and laboratory parameters in hospitalized patients with SARS-CoV2 infection at a community hospital in rural Pennsylvania
Source: PLoS One. 2022 Apr 27;17(4):e0267468. doi: 10.1371/journal.pone.0267468 (PMC9045616; doi:10.1371/journal.pone.0267468)
Supplement: S3 Table — (DOCX) [file pone.0267468.s003.docx]

**S3 Table:** Univariate and Multivariate Logistic regression calculating odds of ventilation for each variable

|  | **Univariate Odds Ratios (95% CI, p-value)** | **Multivariate Odds Ratios (95% CI, p-value)** |
| --- | --- | --- |
| ***Age category (Less than 65)*** | 0.66 (0.30-1.33, p=0.267) | 0.78 (0.09-5.78, p=0.811) |
| ***Gender (Male)*** | 1.65 (0.88-3.19, p=0.127) | 0.61 (0.07-4.55, p=0.628) |
| ***BMI Category*** |  |  |
| Normal | - | - |
| Overweight | 3.82 (0.96-25.57, p=0.092) | 12.67 (0.61-845.12, p=0.148) |
| Obese | 6.97 (1.97-44.32, p=0.010) | 14.96 (1.10-802.35, p=0.092) |
| Severe Obesity | 3.87 (0.88-26.87, p=0.101) | 3.23 (0.04-389.77, p=0.596) |
| ***Smoking status (Yes) ^c^*** | 0.91 (0.49-1.71, p=0.777) | 0.54 (0.08-3.31, p=0.517) |
| ***COPD (Present) ^c^*** | 1.25 (0.54-2.67, p=0.578) | 7.44 (0.91-86.58, p=0.075) |
| ***Diabetes (Present) ^c^*** | 1.54 (0.82-2.88, p=0.177) | 1.46 (0.23-9.08, p=0.680) |
| ***HTN* (Present) ^c^*** | 0.53 (0.28-1.02, p=0.052) | 0.09 (0.01-0.67, p=0.027) |
| ***CHF (CHF) ^c^*** | 0.75 (0.34-1.53, p=0.453) | 1.28 (0.19-9.52, p=0.799) |
| ***CKD (CKD) ^c^*** | 0.92 (0.41-1.89, p=0.829) | 1.43 (0.14-13.24, p=0.750) |
| ***Max Ferritin*** | 1.00 (1.00-1.00, p=0.002) | 1.00 (1.00-1.00, p=0.722) |
| ***Min Hemoglobin**** | 0.72 (0.63-0.83, p<0.001) | 0.71 (0.49-0.97, p=0.044) |
| ***Min Platelets*** | 0.99 (0.98-1.00, p=0.001) | 0.99 (0.97-1.00, p=0.097) |
| ***Max LDH*^,^ ***** | 1.00 (1.00-1.00, p<0.001) | 1.00 (1.00-1.01, p=0.040) |
| ***Max D-Dimer*** | 1.20 (1.11-1.30, p<0.001) | 1.09 (0.90-1.32, p=0.385) |
| ***Minimum lymphocyte count*** | 1.00 (1.00-1.00, p=0.200) | 1.00 (1.00-NA, p=0.230) |
| ***Max WBC**** | 1.20 (1.14-1.27, p<0.001) | 1.31 (1.17-1.53, p<0.001) |
| ***Max CRP*** | 1.13 (1.08-1.18, p<0.001) | 1.09 (1.00-1.22, p=0.070) |
| ***Steroid Use (Yes) ^t^*** | 20.68 (4.41-369.24, p=0.003) | 2617958146.82 (0.00-NA, p=0.991) |
| R***emdesivir (Yes) ^t^*** | 1.53 (0.80-3.08, p=0.212) | 0.42 (0.04-4.67, p=0.474) |

Abbreviations: min: Minimum; max: Maximum; BMI: Body Mass Index; HTN: Hypertension; COPD: Chronic Obstructive Pulmonary Disease; CHF: Congestive Heart Failure; CKD: Chronic Kidney Disease. c: Odds of mechanical ventilation among patients with the comorbidity compared with no comorbidity. t: Odds of mechanical ventilation among patients with treatment compared with no treatment. *: Variables significantly affecting the odds of mechanical ventilation, **: LDH values were divided by 10 and the odds of mechanical ventilation should be interpreted for every 10 unit increase in LDH values. NA: value could not be computed by the software program.
